# Ammonium Alleviates Manganese Toxicity and Accumulation in Rice by Down-Regulating the Transporter Gene *OsNramp5* Through Rhizosphere Acidification

**DOI:** 10.3389/fpls.2019.01194

**Published:** 2019-10-03

**Authors:** An Yong Hu, Man Man Zheng, Li Ming Sun, Xue Qiang Zhao, Ren Fang Shen

**Affiliations:** ^1^School of Geographic Science, Nantong University, Nantong, China; ^2^State Key Laboratory of Soil and Sustainable Agriculture, Institute of Soil Science, Chinese Academy of Sciences, Nanjing, China; ^3^University of Chinese Academy of Sciences, Beijing, China

**Keywords:** ammonium, nitrate, Mn toxicity, pH, Mn transporter gene

## Abstract

Ammonium ($NH_{4}^{+}$) alleviates manganese (Mn) toxicity in various plant species, but the underlying mechanisms are still unclear. In this study, we compared the effects of $NH_{4}^{+}$ and nitrate ($NO_{3}^{-}$) on rice (*Oryza sativa* L.) growth, accumulation and distribution of Mn, accumulation of iron (Fe), zinc (Zn) and copper (Cu), root cell wall components, and expression of Mn and Fe transporter genes. After rice seedlings were grown in non-pH-buffered nutrient solution for 2 days, the pH of growth medium changed from an initial value of 4.5 to 3.5 and to 5.5 in the presence of $NH_{4}^{+}$ and in the presence of $NO_{3}^{-}$, respectively. Compared with $NO_{3}^{-}$, ammonium decreased nutrient-solution pH and alleviated Mn toxicity and accumulation in rice under non-pH-buffered conditions. This alleviation disappeared when 5 mM Homo-PIPES pH buffer was added. Regardless of N form, roots, shoots, root cell sap, and xylem sap accumulated much lower Mn at pH 3.5 than at pH 5.5, whereas Mn distribution in different leaves and Mn accumulation in root cell walls was affected by neither N form nor pH. Ammonium decreased the expression of the Mn influx transporter gene *OsNramp5* in roots under non-pH-buffered conditions, but not under pH-buffered ones. *OsNramp5* expression was down-regulated at pH 3.5 compared with pH 5.5. Another efflux Mn transporter gene, Os*MTP9*, was not regulated by either N form or pH. High pH (5.5) enhanced the expression of the Fe transporter gene *OsIRT1* and increased the accumulation of Zn but not Fe or Cu in shoots compared with pH 3.5. Taken together, our results indicate that $NH_{4}^{+}$ alleviates Mn toxicity and accumulation in rice through the down-regulatory effects of rhizosphere acidification on the Mn influx transporter gene *OsNramp5*. In addition, the up-regulation of *OsIRT1* expression may contribute to the increased Zn uptake by rice at high pH of nutrient solution.

## Introduction

Manganese (Mn), an essential microelement required by plants, is involved in the regulation of several metabolic processes, such as photosynthesis, respiration, and antioxidant activity. As a cofactor, Mn plays an important role in activating approximately 35 different enzymes (Broadley et al., 2012). Excess Mn can be toxic to plant growth, however, especially in acid soils where Mn availability is increased because of soil acidification. Mn toxicity may be the second most important limiting factor, after aluminum (Al) toxicity, for plants in acid soils (Foy, 1984; Millaleo et al., 2010). Approximately 30% of the world’s ice-free land and 50% of arable and potentially arable lands are composed of acid soils (Von Uexküll and Mutert, 1995). Low pH (<5.5) and reducing-soil conditions caused by excess water or poor drainage can greatly increase the concentration of soluble Mn^2+^ in soils, resulting in Mn toxicity to plants (Chesworth, 1991; Watmough et al., 2007; Huang et al., 2015). Depending on plant species, the threshold of Mn accumulation before Mn toxicity occurs generally varies from 200 to 3,500 mg Mn·kg^−1^ DW (Krämer, 2010), but some Mn hyperaccumulators, such as *Proteaceae*, *Phytolacca*, and *Gossia* species, can accumulate more than 10,000 mg Mn·kg^−1^ DW in their aerial parts without detrimental effects (Brooks et al., 1981; Xue et al., 2004; Fernando et al., 2009; Liu et al., 2010).

Nitrogen (N) is an essential macroelement required by plants and is taken up by roots in two main forms, ammonium ($NH_{4}^{+}$) and nitrate ($NO_{3}^{-}$) (Li et al., 2013). In acid soils, the predominant inorganic form of N is $NH_{4}^{+}$ because such soils have a weak nitriﬁcation capacity due to low pH and the application of $NH_{4}^{+}$-N fertilizers (De Boer and Kowalchuk, 2001; Che et al., 2015). Previous studies have found that $NH_{4}^{+}$ alleviates Mn toxicity and/or decreases the concentration of Mn compared with $NO_{3}^{-}$ in barley (*Hordeum vulgare*) (Arnon, 1937; Vlamis and Williams, 1962), *Holcus lanatus* and *Bromus erectus* (McGrath and Rorison, 1982), muskmelon (*Cucumis melo* L.) (Elamin and Wilcox, 1986), marigold (*Tagetes erects* L. and *T. patula* L.) (Reddy and Mills, 1991), and Norway spruce (*Picea abies* L. Karst.) (Langheinrich et al., 1992). Although the alleviatory effects of $NH_{4}^{+}$ on Mn toxicity may be attributed to the antagonism of $NH_{4}^{+}$ on Mn^2+^ uptake (McGrath and Rorison, 1982), the exact mechanisms are unclear.

Rice (*Oryza sativa* L.) is an important staple food crop consumed by nearly half of the world’s population (Muthayya et al., 2014). Approximately 13% of global rice production occurs in acid soils (Von Uexküll and Mutert, 1995). Rice has traditionally been cultivated in anaerobic paddy soils, where the predominant N source is $NH_{4}^{+}$-N. Rice plants are thus considered to prefer $NH_{4}^{+}$ over $NO_{3}^{-}$ (Zhao et al., 2014; Zhao and Shen, 2018). Rice is also one of the most Mn-tolerant crops (Lidon, 2001; Chen et al., 2013). Compared with other grasses, rice can accumulate 5–10 times more leaf Mn while showing milder symptoms of Mn toxicity (Foy et al., 1978). Rice can tolerate up to 5 g Mn·kg^−1^ dry weight in the leaves without showing any toxic symptoms, whereas barley suffers from Mn toxicity at accumulations of only 150 mg Mn·kg^−1^ dry weight (Vlamis and Williams, 1964). Despite these observations, relevant studies on the effects of the form of N on Mn toxicity in rice and the underlying mechanisms are still lacking. In the present study, we therefore investigated the effect of $NH_{4}^{+}$ and $NO_{3}^{-}$ on Mn toxicity in rice and further explored potential physiological and molecular mechanisms.

## Materials and Methods

### Plant Materials and Growth Conditions

We used *O. sativa* ssp. *japonica* “Nipponbare” in this study. Seeds were soaked in deionized water at 30°C for 2 days in the dark and then transferred to a net floating on a 0.5mM CaCl_2_ solution (pH 4.5) in a black plastic container. After 7 days, uniform seedlings were transferred to a 3.2-L black pot containing nutrient solution and cultivated in a controlled environmental growth chamber under a 14-h/10-h (30°C/25°C) day–night cycle and a relative humidity of 65% for 7 or 19 days. The nutrient solution was a modified full-strength Kimura B solution (pH 4.5) containing 0.5 mM NH_4_NO_3_, 0.18 mM KH_2_PO_4_, 0.55 mM KCl, 0.54 mM MgSO_4_·7H_2_O, 0.36 mM CaCl_2_·2H_2_O, 0.5 µM MnCl_2_·4H_2_O, 3 µM H_3_BO_3_, 1 µM NaMoO_4_·2H_2_O, 0.4 µM ZnSO_4_·7H_2_O, 0.2 µM CuSO_4_·5H_2_O, and 20 µM Fe(III)-EDTA. The solution was renewed every 2 days. Rice seedlings (14 or 26 days old) were treated with 1 mM $NH_{4}^{+}$ or 1 mM $NO_{3}^{-}$ at different Mn concentrations and pHs under non-pH-buffered or pH-buffered conditions as described below. $NH_{4}^{+}$ and $NO_{3}^{-}$ were supplied as NH_4_Cl and NaNO_3_, respectively. Manganese was supplied as MnCl_2_·4H_2_O. All experiments were conducted with three to four biological replicates.

### Experimental Treatments Under Non-pH-Buffered Conditions

Rice seedlings (14 days old) were grown in a modified full-strength Kimura B nutrient solution (pH 4.5) containing 1 mM $NH_{4}^{+}$ or 1 mM $NO_{3}^{-}$ with different Mn concentrations depending on the experiments. After treated with 0.5 or 500 µM Mn^2+^ in different N forms for 1 and 3 days, the roots were sampled for the expression analysis of Mn transporter genes. The solution was renewed after 1.5 days. After treated with 0.5 or 500 µM Mn^2+^ in different N forms for 10 days, the roots were used for the extraction and measurement of cell wall polysaccharide fractions. After treated with 0.5, 200, 500, or 1,000 µM Mn^2+^ in different N forms for 14 days, the roots were washed three times with cold 5 mM CaCl_2_ solution and separated from the shoots for dry weight measurements and Mn determination. The solution was renewed every 2 days.

### Experimental Treatments Under pH-Buffered Conditions

Rice seedlings (14 days old) were cultured in a modified full-strength Kimura B nutrient solution containing 0.5 or 500 µM Mn^2+^ with 1 mM $NH_{4}^{+}$ or 1 mM $NO_{3}^{-}$ at a pH of 3.5 or 5.5 buffered with 5 mM homopiperazine-1,4-bis(2-ethanesulfonic acid) (Homo-PIPES). After 3 days, the roots were washed three times with cold 5 mM CaCl_2_ solution and separated from the shoots for Mn, Fe, Zn, and Cu determination. The roots were also sampled for the expression analysis of Mn and Fe transporter genes.

Rice seedlings (26 days old) were cultured in a modified full-strength Kimura B nutrient solution containing 500 µM Mn^2+^ with 1 mM $NH_{4}^{+}$ or 1 mM $NO_{3}^{-}$ at a pH of 3.5 or 5.5 buffered with 5 mM Homo-PIPES. After 24 h, root cell sap, cell walls, and xylem Sap were collected. After 3 days, the roots and each individual leaf (leaf 2–10, from old to young) were sampled for dry weight and Mn determination. Leaf 1 was too small to be sampled.

In the above experiments, the nutrient solution was adjusted to a pH of 3.5 or 5.5 every 12 h by addition of 0.1 M HCl or NaOH and renewed at 36 h.

### Collection of Root Cell Sap, Cell Walls, and Xylem Sap

The shoots (2 cm above the root) were decapitated with a razor, and xylem sap was collected with a micropipette for 1 h. The excised roots were placed in a filter with 0.45 µm microporous membrane in a tube (Millipore, Billerica, MA, USA) and frozen at −80°C overnight. After thawing to room temperature, root cell sap was obtained by centrifugation at 20,600 ×*g* for 10 min. The residual cell walls were washed three times with 70% ethanol every 5 min and then dried at 70°C for 3 days. Mn concentrations in root cell sap, cell walls, and xylem sap were determined as described below.

### Determination of Mn, Fe, Zn, and Cu

Dried samples were digested with concentrated HNO_3_ at 140°C. After appropriate dilution, the concentration of Mn and Fe in roots and shoots was determined by Inductive Coupled Plasma Optical Emission Spectroscopy (ICP-OES) (Optima8000; Perkin-Elmer, Waltham, Massachusetts, USA), and that of Zn and Cu was determined by Inductive Coupled Plasma Mass Spectroscopy (ICP-MS) (7700X; Agilent Technologies, Santa Clara, California, USA). The Mn concentration in root cell sap, xylem sap, and digested cell walls was also determined by ICP-MS.

### Expression Analysis of Mn and Fe Transporter Genes

Total RNA from rice roots was extracted using an RNeasy Plant Mini kit (Qiagen) and converted to cDNA using ReverTra Ace qPCR RT Master Mix with gDNA Remover (Toyobo) following the manufacturer’s protocols. The quantitative PCR was performed on a LightCycler 480 Instrument (Roche, Switzerland) using SYBR premix Ex *Taq* (Takara). Primer sequences used for amplification of *OsNramp5* were 5’-CAGCAGCAGTAAGAGCAAGATG-3’ (forward) and 5’-GTGCTCAGGAAGTACATGTTGAT-3’ (reverse); 5’-AGGACCATTTCTTCGACGTG-3’ (forward) and 5’-TCCATCCACCATTTGTACCG-3’ (reverse) for *OsMTP9*; 5’-CGTCTTCTTCTTCTCCACCACGAC-3’ (forward) and 5’-GCAGCTGATGATCGAGTCTGACC-3’ (reverse) for *OsIRT1*. *HistoneH3* was used as an internal standard with primers pairs 5’-AGTTTGGTCGCTCTCGATTTCG-3’ (forward) and 5’-TCAACAAGTTGACCACGTCACG-3’ (reverse). The relative expression was normalized by the ΔΔ Ct method.

### Extraction and Measurement of Cell Wall Polysaccharide Fractions in Roots

Extraction of cell walls was conducted according to Zhong and Läuchli (1993). First, roots were homogenized in 8 ml of 75% ethanol for 20 min and centrifuged at 3,400 × g for 10 min. The resulting pellets (cell walls) were eluted in 8 ml acetone, 1:1 methanol/chloroform, and then methanol for 20 min each and then dried at 60°C. The cell wall material was weighed (∼2 mg) and incubated three times with 1 ml ultrapure water in boiling water for 1 h. The supernatants were collected as the pectin fraction. The residues were extracted twice with 1 ml of 24% KOH containing 0.02% KBH_4_ at room temperature, and the supernatants were used as the hemicellulose fraction. In accordance with previous studies, the uronic acid content of the pectin and hemicellulose fractions was determined spectrophotometrically at 520 and 490 nm, respectively (Dubois et al., 1956; Blumenkrantz and Asboe-Hansen, 1973).

### Statistical Analysis

All experiments were repeated independently at least two times with three to four biological replicates, and a representative set of data is presented in the *Results*. The significance of differences between means (at *P* < 0.05) was analyzed by Duncan’s test.

## Results

### Ammonium Alleviated Mn Toxicity and Decreased Mn Uptake by Rice Compared With $NO_{3}^{-}$

**Figure 1:**
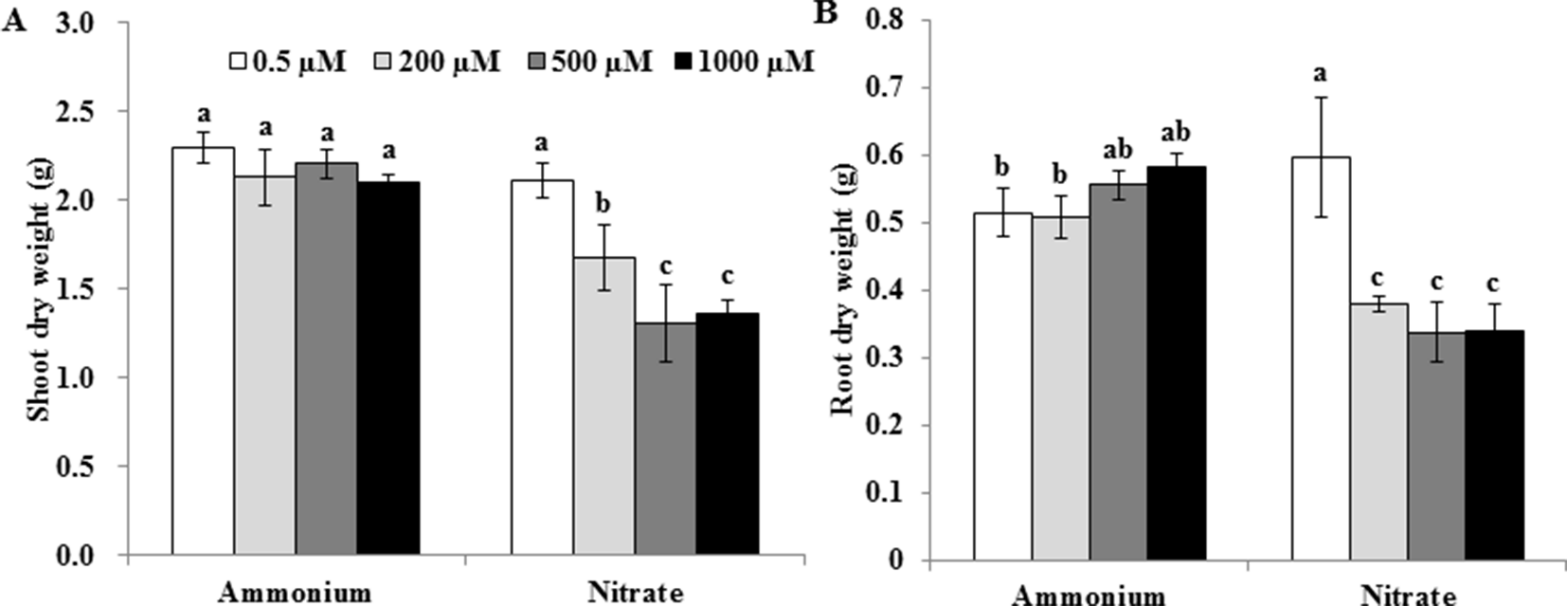
Effect of different N forms on the growth of rice exposed to various concentrations of Mn under non-pH-buffered conditions. **(A)** Shoot dry weight; **(B)** root dry weight. Rice seedlings (14 days old) were cultured in a modified Kimura B nutrient solution (pH 4.5) containing 1 mM ammonium or nitrate with different Mn concentrations for 14 days. Data represent means ± SD (*n* = 3). Different lowercase letters indicate a significant difference (*P* < 0.05) based on Duncan’s test.

At a Mn concentration of 0.5 µM, shoot dry weights were similar under $NH_{4}^{+}$ and $NO_{3}^{-}$ treatments (**Figure 1A**), whereas root dry weights were lower under $NH_{4}^{+}$ than under $NO_{3}^{-}$ (**Figure 1B**). At Mn concentrations of 200, 500, and 1,000 µM, however, dry weights of both roots and shoots were higher under $NH_{4}^{+}$ than under $NO_{3}^{-}$ (**Figures 1A, B**). Increasing the Mn concentration did not decrease dry weights of shoots and roots under $NH_{4}^{+}$ (**Figures 1A, B**). Compared with 0.5 µM Mn, however, dry weights of shoots and roots under $NO_{3}^{-}$ decreased when rice plants were treated with 200, 500 or 1,000 µM Mn (**Figures 1A, B**).

**Figure 2:**
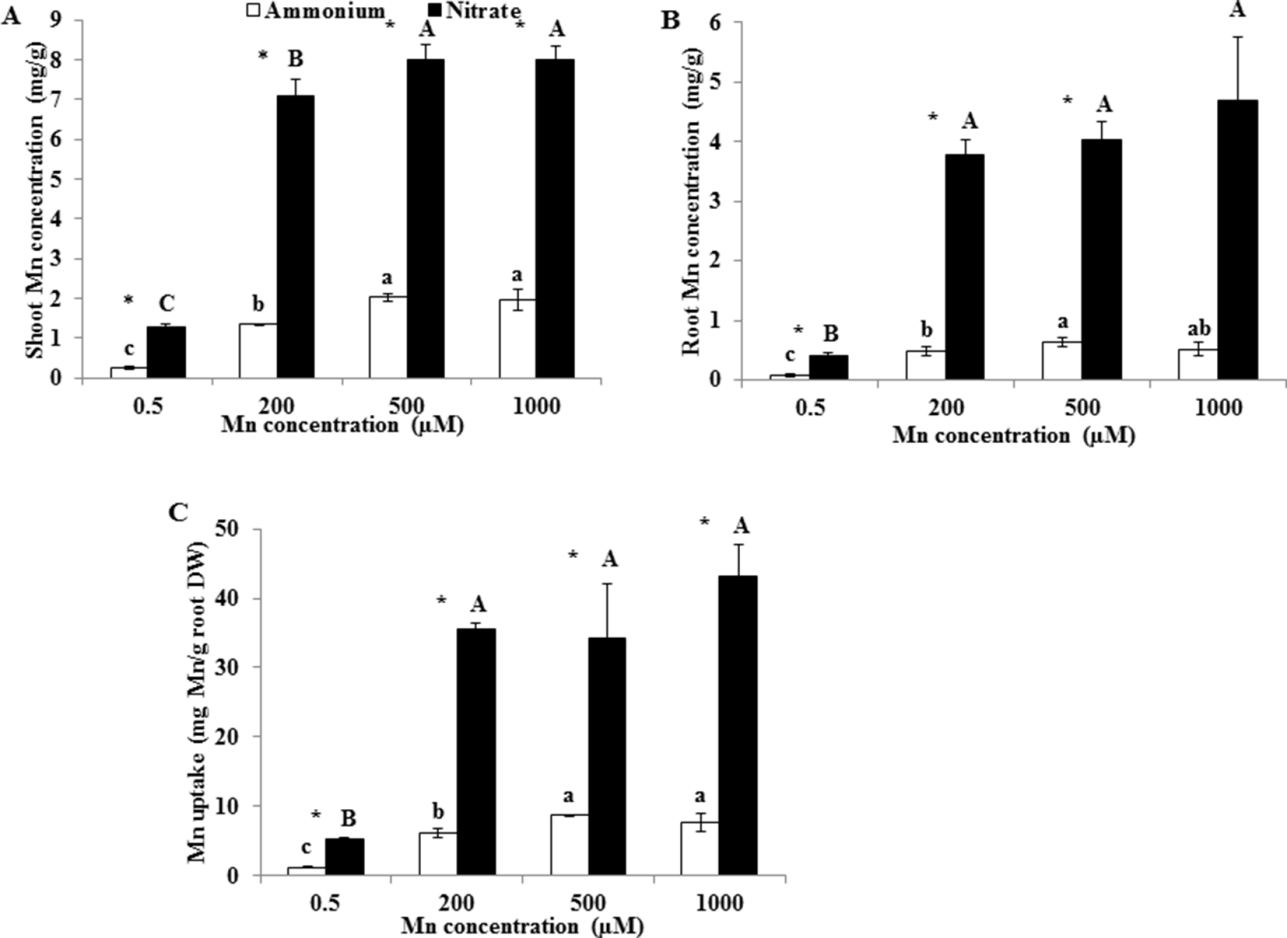
Effect of different N forms on Mn accumulation in rice under various concentrations of Mn under non-pH-buffered conditions. **(A**–**B)** Mn concentration in shoots **(A)** and roots **(B)**. **(C)** Mn uptake (mg Mn/g root DW). Mn uptake was calculated as the total Mn content in shoots and roots per root dry weight. Rice seedlings (14 days old) were cultured in a modified Kimura B nutrient solution (pH 4.5) containing 1 mM ammonium or nitrate with different Mn concentrations for 14 days. Data represent means ± SD (*n* = 3). Different lowercase letters above white columns indicate significant differences (*P* < 0.05, Duncan’s test) among different Mn concentrations under the ammonium-N treatment; different uppercase letters above black columns indicate significant differences (*P* < 0.05, Duncan’s test) among different Mn concentrations under the nitrate-N treatment. An asterisk indicates a significant difference (*P* < 0.05, Student’s *t*-test) between ammonium and nitrate treatments at the same Mn concentration.

Root and shoot Mn concentrations and Mn uptake were remarkably higher under $NO_{3}^{-}$ than under $NH_{4}^{+}$ at either Mn treatment concentration (**Figure 2**). Regardless of the form of N, shoot Mn concentrations increased between 0.5 and 500 µM Mn treatments, but not between 500 and 1,000 µM Mn (**Figure 2A**). Under both $NH_{4}^{+}$ and $NO_{3}^{-}$ treatments, root Mn concentrations greatly increased between 0.5 and 200 µM Mn treatments, but not between 200 and 1,000 µM Mn (**Figure 2B**). Mn uptake increased until the 500 µM Mn treatment under $NH_{4}^{+}$ and until the 200 µM Mn treatment under $NO_{3}^{-}$ (**Figure 2C**).

### Low pH Decreased Rice Mn Uptake But Did Not Affect Mn Distribution in Rice Leaves

**Figure 3:**
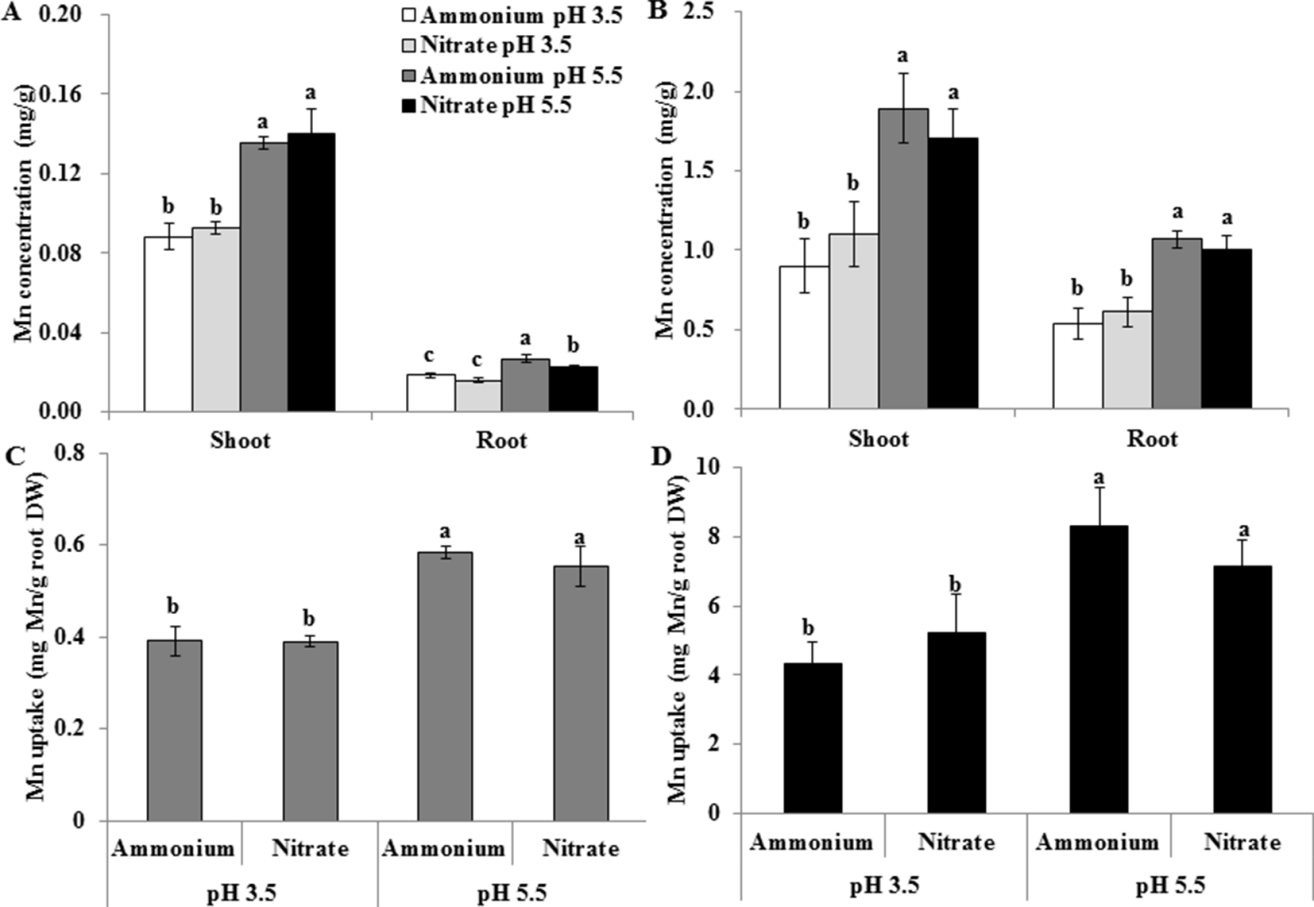
Effect of different pH levels and N forms on Mn accumulation in rice under pH-buffered conditions. **(A**–**B)** Mn concentration under 0.5 µM Mn **(A)** and 500 µM Mn **(B)** treatments. **(C**–**D)** Mn uptake (mg Mn/g root DW) under 0.5 µM Mn **(C)** and 500 µM Mn **(D)** treatments (total Mn/root DW). Mn uptake was calculated as the total Mn content in shoots and roots per root dry weight. Rice seedlings (14 days old) were cultured in a modified Kimura B nutrient solution containing 1 mM ammonium or nitrate with 0.5 or 500 µM Mn at pH 3.5 or 5.5 for 3 days. The pH of the nutrient solution was buffered with 5 mM Homo-PIPES. Data represent means ± SD (*n* = 3). Different lowercase letters indicate a significant difference (*P* < 0.05) based on Duncan’s test

In the above experiment, the nutrient solution pH decreased from an initial value of 4.5 to 3.5 in the presence of $NH_{4}^{+}$ before renewal of the nutrient solution and increased from 4.5 to 5.5 in the presence of $NO_{3}^{-}$. We therefore maintained the pH of nutrient solution at 3.5 and 5.5 by buffering with 5 mM Homo-PIPES and further investigated whether the changes of the pH of the solutions due to uptake of $NH_{4}^{+}$ and $NO_{3}^{-}$ help regulate the effects of the two forms of N on Mn uptake by rice. Regardless of the N form and the Mn concentration of the nutrient solution, Mn concentrations of roots and shoots and Mn uptake were always higher at pH 5.5 than at pH 3.5 (**Figure 3**). At a given pH, however, no difference was observed in the Mn concentration of roots and shoots and Mn uptake between $NH_{4}^{+}$ and $NO_{3}^{-}$ (**Figure 3**).

**Figure 4:**
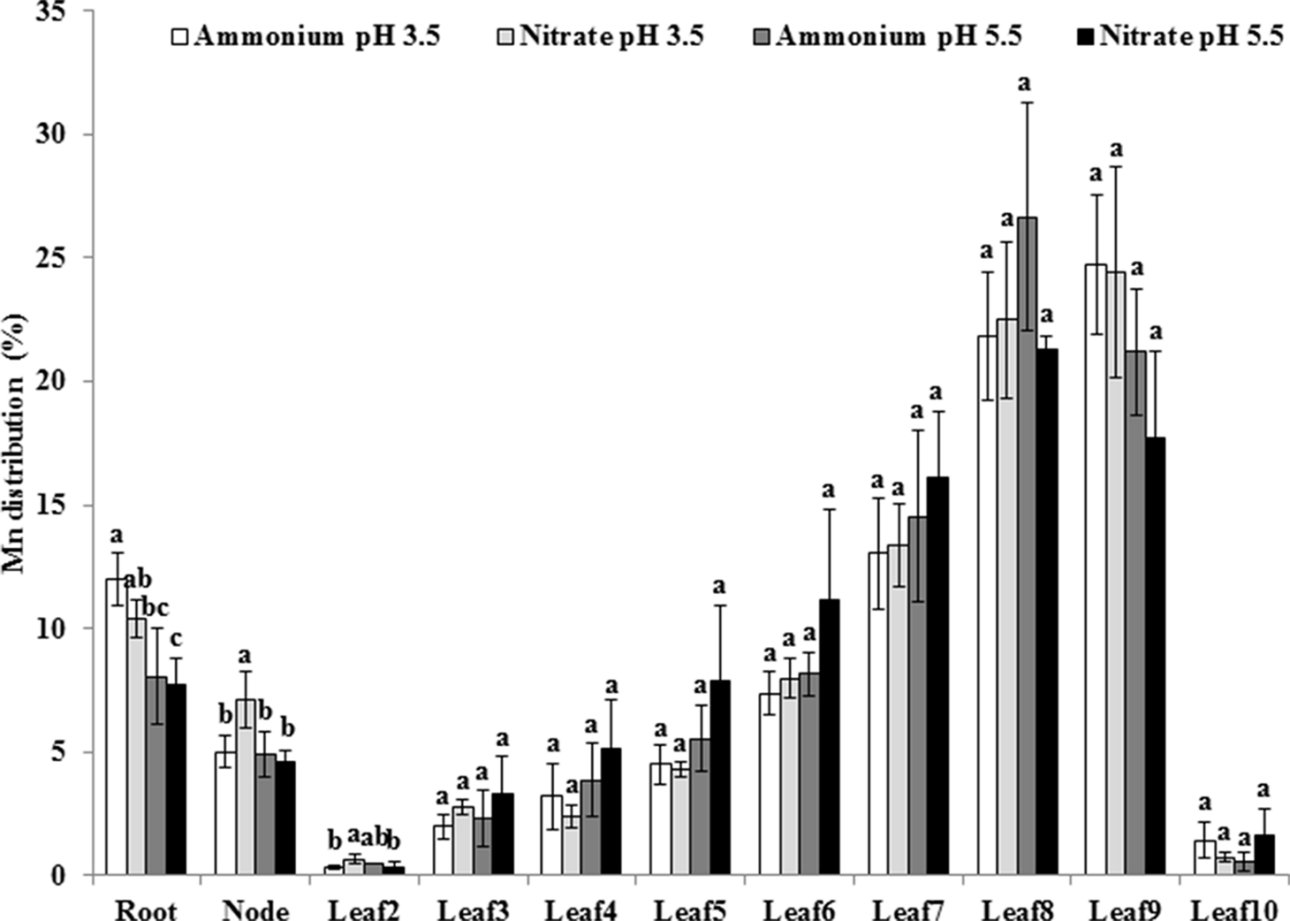
Effect of different N forms and pH levels on Mn distribution in different rice organs under pH-buffered conditions. Rice seedlings (26 days old) were cultured in a modified Kimura B nutrient solution containing 500 µM Mn with 1 mM ammonium or nitrate at pH 3.5 or 5.5 for 3 days. The pH of the nutrient solution was buffered with 5 mM Homo-PIPES. Data represent means ± SD (*n* = 3). Different lowercase letters indicate a significant difference (*P* < 0.05) based on Duncan’s test.

To examine whether pH affects Mn distribution to different organs, we determined Mn concentrations of different organs and calculated distribution ratios at pH levels of 3.5 and 5.5 in the presence of $NH_{4}^{+}$ or $NO_{3}^{-}$. Approximately 10% of total Mn was distributed in the roots, while 5% was present in the nodes (**Figure 4**). The Mn distribution ratio in roots was higher at pH 3.5 than at pH 5.5 under the same N form (**Figure 4**). In aerial parts of rice plants, the majority of Mn was located in young leaves except for leaf 10, which had not yet fully expanded, but no difference was detected in Mn distribution of the leaves between pH 3.5 and pH 5.5 and between $NH_{4}^{+}$ and $NO_{3}^{-}$ (**Figure 4**).

### Low pH Decreased Mn Concentrations in Rice Root Cell Sap and Xylem Sap, But Not Root Cell Walls

**Figure 5:**
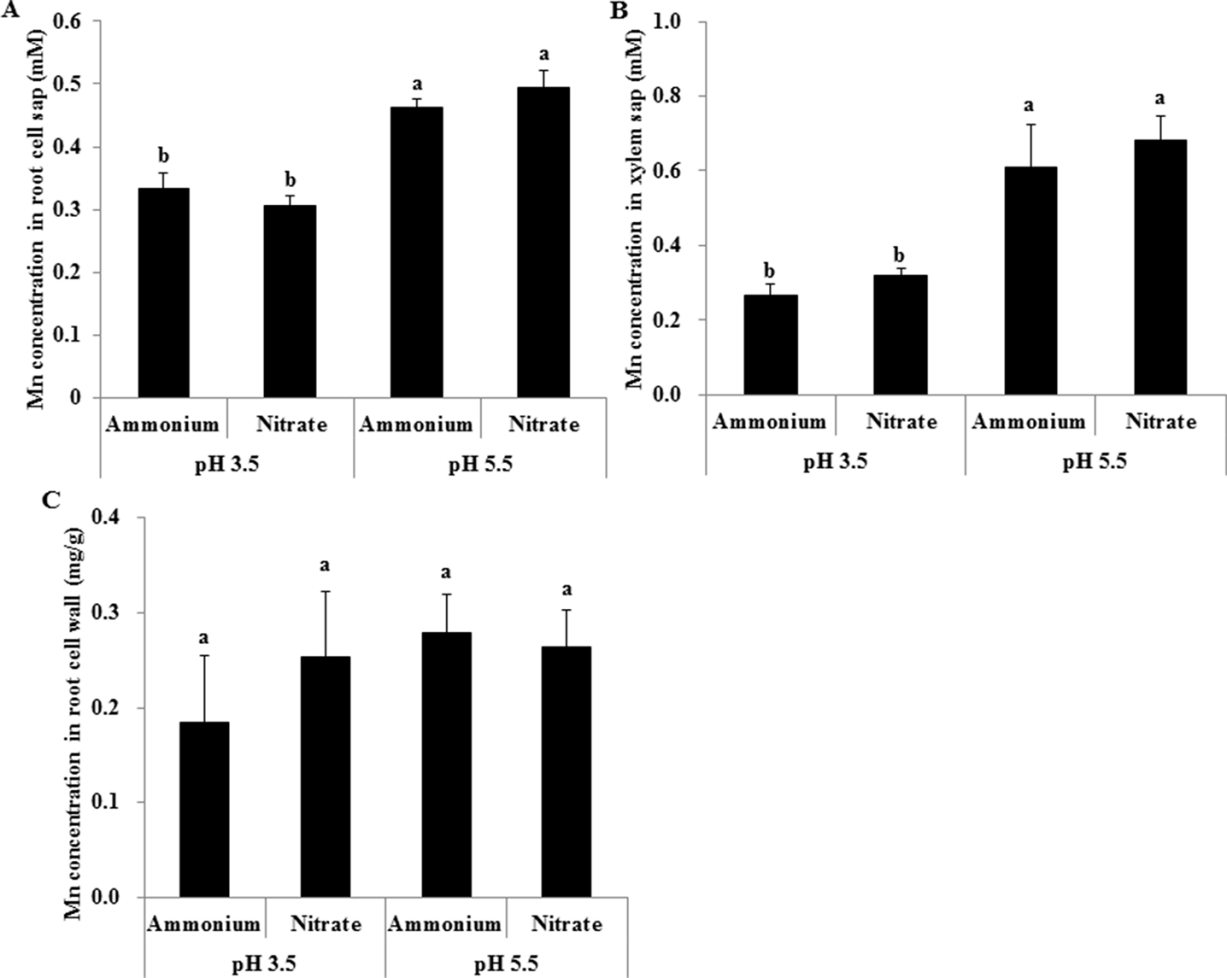
Effect of different pH levels on Mn concentration in xylem sap, root cell sap, and cell walls under pH-buffered conditions. **(A**–**C)** Mn concentration in root cell sap **(A)**, xylem sap **(B)**, and root cell walls **(C)**. Rice seedlings (26 days old) were cultured in a modified Kimura B nutrient solution containing 1 mM ammonium or nitrate with 500 µM Mn at pH 3.5 or 5.5 for 24 h. The pH of the nutrient solution was buffered with 5 mM Homo-PIPES. Data represent means ± SD (*n* = 4). Different lowercase letters indicate a significant difference (*P* < 0.05) based on Duncan’s test.

Mn concentrations in root cell sap and xylem sap were much higher at pH 5.5 than at pH 3.5 regardless of N form, but were similar between $NH_{4}^{+}$ and $NO_{3}^{-}$ treatments at the same pH (**Figures 5A, B**). Neither pH nor the form of N had an effect on Mn concentrations in root cell walls (**Figure 5C**).

### Low pH Decreased the Expression of the Mn Transporter Gene *OsNramp5*, But Not *OsMTP9*, in Rice Roots

**Figure 6:**
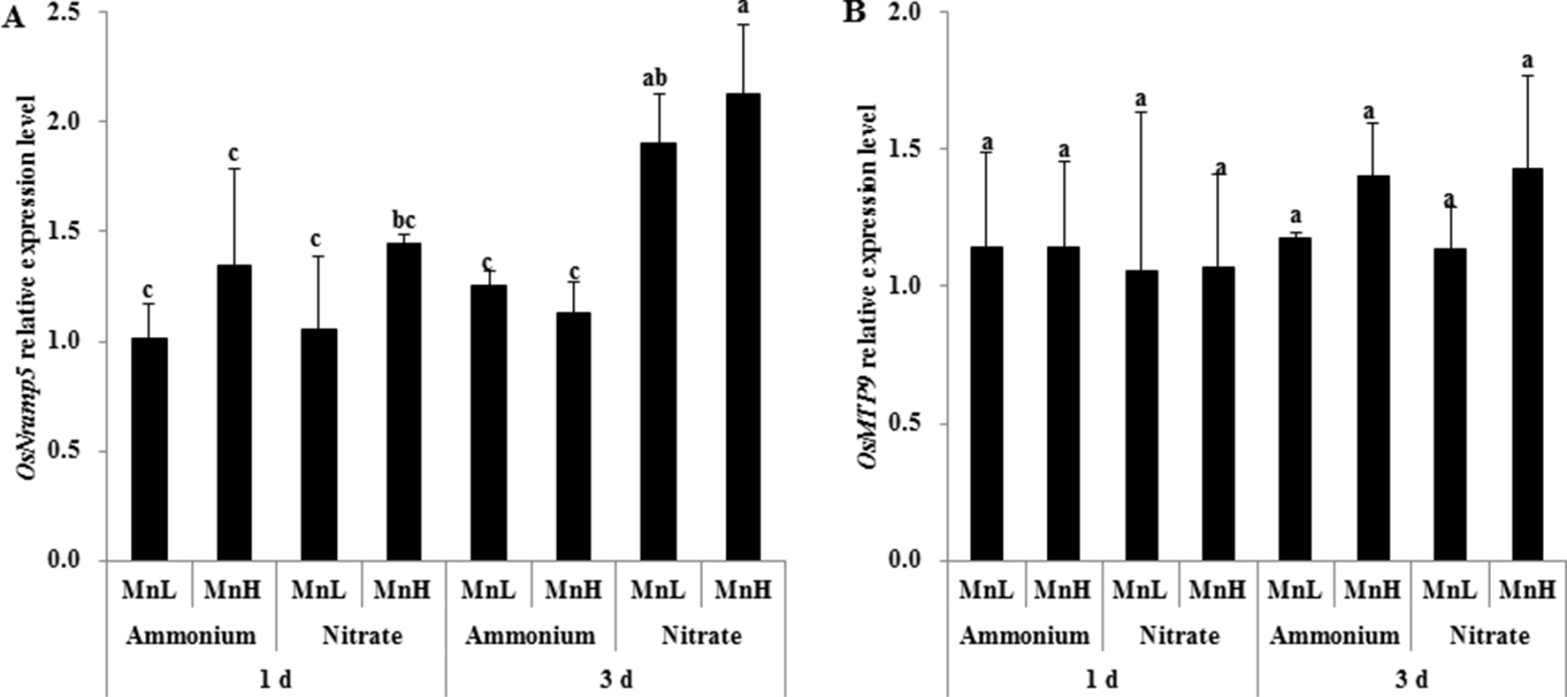
Effect of different N forms and pH levels on the expression of Mn transporter genes under non-pH-buffered conditions. **(A)** OsNramp5; **(B)** OsMTP9. Rice seedlings (14 days old) were cultured in 0.5 µM Mn (MnL) or 500 µM Mn (MnH) in the presence of 1 mM ammonium or nitrate with a non-pH-buffered solution (pH 4.5) for 1 and 3 days. The roots were sampled for RNA extraction. Data represent means ± SD (*n* = 3). Different lowercase letters indicate a significant difference (*P* < 0.05) based on Duncan’s test.

**Figure 7:**
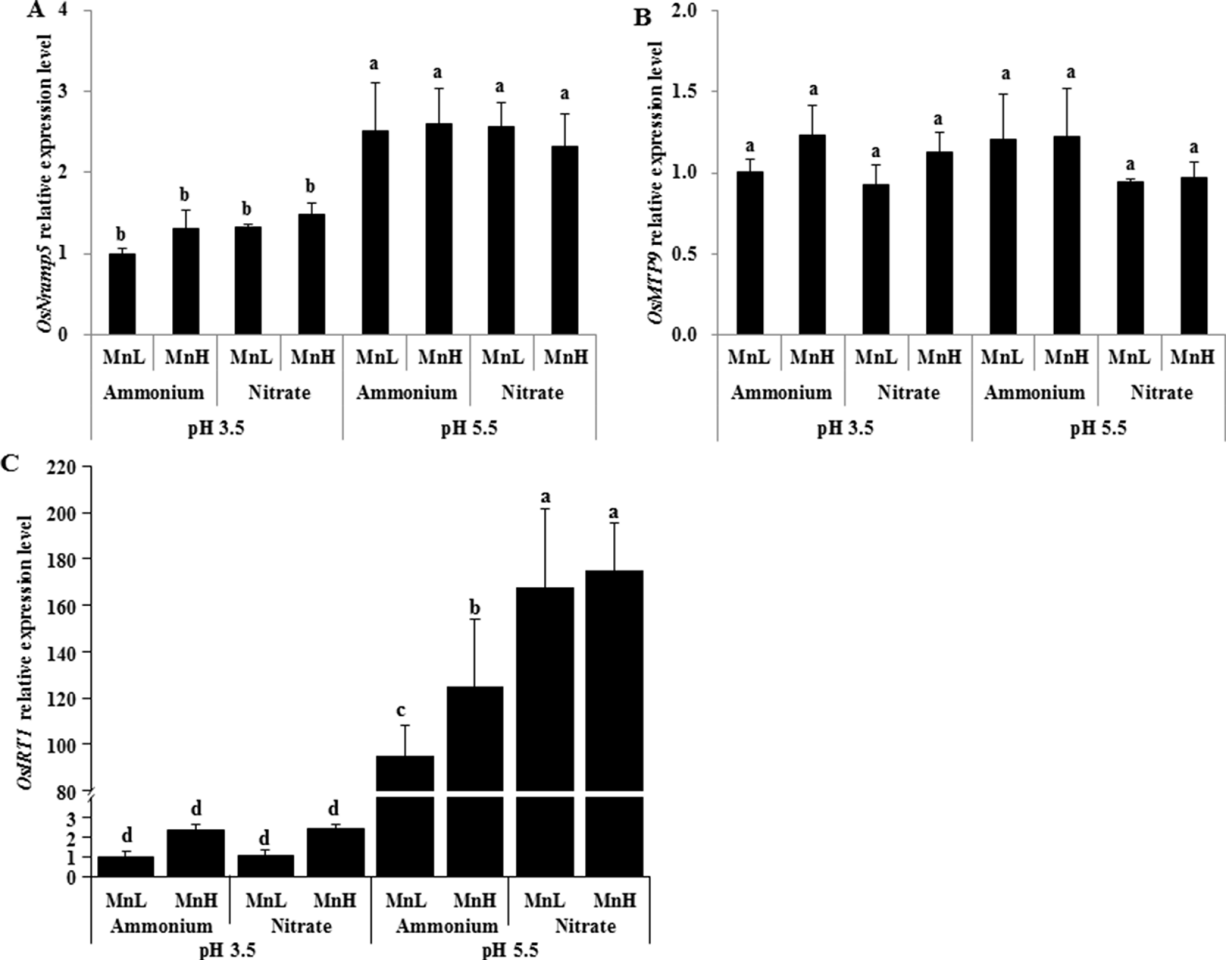
Effect of different N forms and pH levels on the expression of Mn and Fe transporter genes under pH-buffered conditions. **(A)** OsNramp5; **(B)** OsMTP9; **(C)** OsIRT1. Rice seedlings (14 days old) were cultured in 0.5 µM Mn (MnL) or 500 µM Mn (MnH) in the presence of 1 mM ammonium or nitrate at a pH of 3.5 or 5.5 buffered with 5 mM Homo-PIPES for 3 days. The roots were sampled for RNA extraction. Data represent means ± SD (*n* = 4). Different lowercase letters indicate a significant difference (*P* < 0.05) based on Duncan’s test.

Rice Mn uptake is mediated by two known transporters, OsNramp5 and OsMTP9 (Ishimaru et al., 2012; Sasaki et al., 2012; Ueno et al., 2015). We therefore investigated expression levels of the two Mn transporter genes, *OsNramp5* and *OsMTP9*, in roots exposed to non-pH-buffered or buffered solutions with different N forms and Mn concentrations. After 1-day exposure, the expression level of *OsNramp5* was similar between $NH_{4}^{+}$ and $NO_{3}^{-}$ at both Mn concentrations (**Figure 6A**). After 3-day exposure, the expression level of *OsNramp5* was up-regulated by $NO_{3}^{-}$ compared with $NH_{4}^{+}$ (**Figure 6A**). No difference was detected in the expression level of *OsMTP9* between $NH_{4}^{+}$ and $NO_{3}^{-}$ after either 1 or 3 days of exposure (**Figure 6B**). The expression level of *OsNramp5* was up-regulated at pH 5.5 relative to pH 3.5 buffered with 5 mM Homo-PIPES regardless of N form and Mn concentration after 3-day exposure, but was unaffected by the form of N at a given pH (**Figure 7A**). *OsMTP9* expression was unaffected by pH and N form under pH-buffered conditions (**Figure 7B**). At a given pH or exposure to the same N form, the Mn concentration of the growth medium did not affect the expression of either *OsNramp5* or *OsMTP9* (**Figures 6** and **7**).

### Low pH Decreased the Expression of the Fe Transporter Gene *OsIRT1* and Zn Uptake by Rice

**Figure 8:**
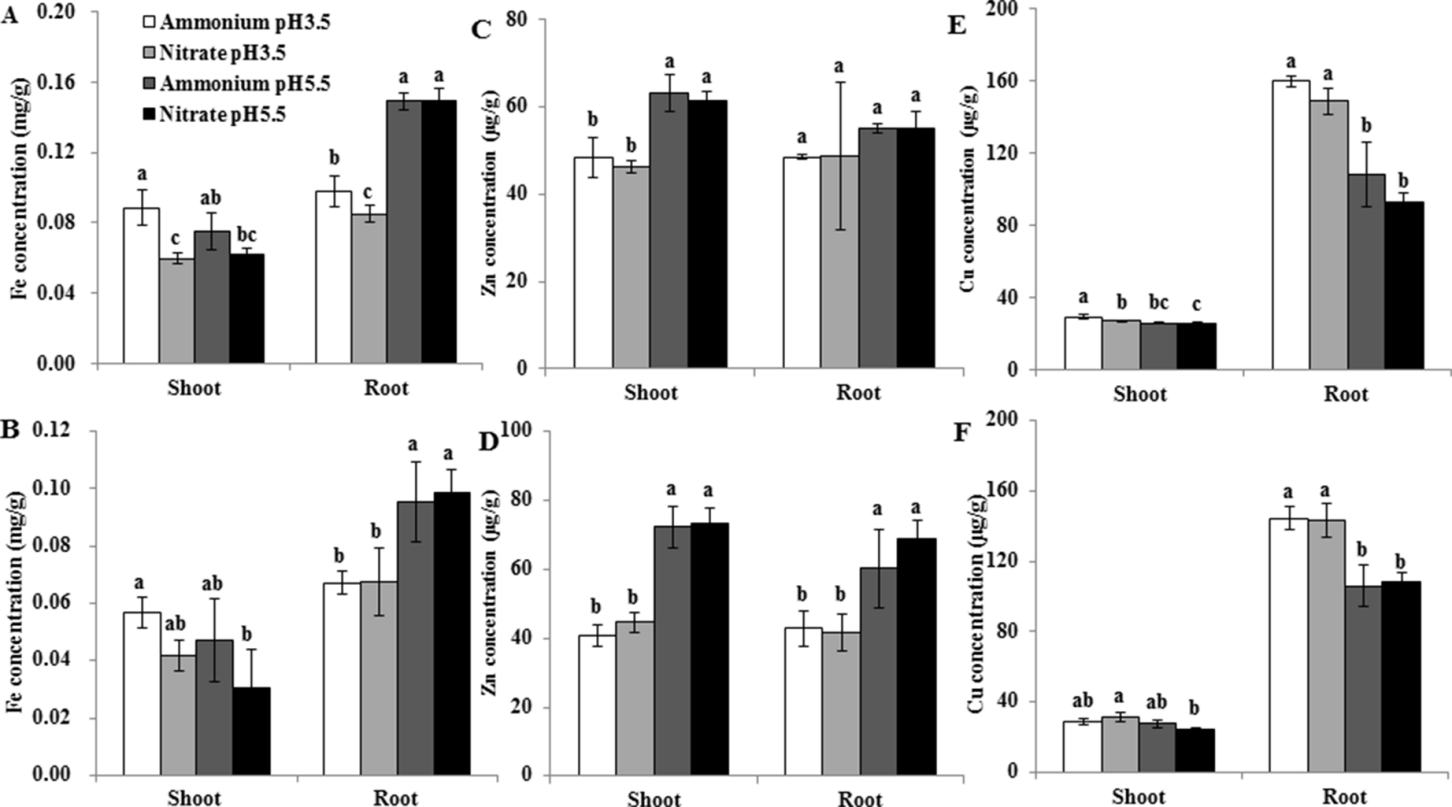
Effect of different pH levels and N forms on the accumulation of Fe, Zn, and Cu in rice under pH-buffered conditions. **(A**–**F)** Concentration of Fe **(A**, **B)**, Zn **(C**,**D)**, and Cu **(E**,**F)** in the roots and shoots under 0.5 µM Mn **(A**,**C**,**E)** and 500 µM Mn **(B**,**D**,**F)** treatments. Rice seedlings (14 days old) were cultured in a modified Kimura B nutrient solution containing 1 mM ammonium or nitrate with 0.5 or 500 µM Mn at pH 3.5 or 5.5 for 3 days. The pH of the nutrient solution was buffered with 5 mM Homo-PIPES. Data represent means ± SD (*n* = 3). Different lowercase letters indicate a significant difference (*P* < 0.05) based on Duncan’s test.

OsNramp5 can also transport Fe in rice (Ishimaru et al., 2012; Sasaki et al., 2012). OsIRT1 was demonstrated to be involved in the uptake of Fe and Zn (Ishimaru et al., 2006; Lee and An, 2009). In order to investigate whether the effects of pH on Mn uptake and transporter gene expression are specific, we further examined the expression of the Fe transporter gene *OsIRT1* in roots and the accumulation of Fe, Zn, and Cu in rice. The expression level of *OsIRT1* was up-regulated at pH 5.5 relative to pH 3.5, and was up-regulated under $NO_{3}^{-}$ relative to $NH_{4}^{+}$ at pH 5.5 but not at pH 3.5 (**Figure 7C**). The Fe concentration of roots and shoots was higher under $NH_{4}^{+}$ than under $NO_{3}^{-}$ at pH 3.5 with 0.5 µM Mn (**Figure 8A**), but there was no significant difference between $NH_{4}^{+}$ and $NO_{3}^{-}$ under other conditions (**Figures 8A, B**). Low pH decreased the Fe concentration of roots but not shoots at both Mn concentrations (**Figures 8A, B**). Low pH decreased the Zn concentration of shoots at both Mn concentrations, and that of roots at only 500 µM Mn (**Figures 8C, D**). In contrast, low pH increased the Cu concentration of roots but not shoots (**Figures 8E, F**). Less difference was detected in the Cu and Zn concentrations of shoots and roots between $NH_{4}^{+}$ and $NO_{3}^{-}$ at a given pH (**Figures 8C–F**).

## Discussion

### $NH_{4}^{+}$-Induced Rhizosphere Acidiﬁcation Was Responsible for the Decreased Mn Accumulation in Rice

In agreement with previous studies of other plant species (Arnon, 1937; Vlamis and Williams, 1962; McGrath and Rorison, 1982; Elamin and Wilcox, 1986; Reddy and Mills, 1991; Langheinrich et al., 1992), we found that $NH_{4}^{+}$ alleviated Mn toxicity and decreased Mn accumulation in rice. The uptake of $NH_{4}^{+}$ by roots resulted in a decrease in the pH of the growth medium because of the release of protons (Wang et al., 1993; Schubert and Yan, 1997; Zhao et al., 2009). In contrast, the uptake of $NO_{3}^{-}$ by plants alkalized the rhizosphere as a result of the co-transport of H^+^ into cells (Marschner and Römheld, 1983; Moorby et al., 1985; Mistrik and Ullrich, 1996). The pH may be involved in the effects of $NH_{4}^{+}$ and $NO_{3}^{-}$ on Mn toxicity and uptake by rice. When 5 mM Homo-PIPES was used to maintain the pH of the growth medium, no difference was observed in rice Mn accumulation between $NH_{4}^{+}$ and $NO_{3}^{-}$. Remarkably, Mn accumulation in rice was much lower at pH 3.5 than pH 5.5. The results of these analyses suggest that $NH_{4}^{+}$-induced rhizosphere acidiﬁcation was responsible for the decreased Mn accumulation in rice fed with $NH_{4}^{+}$ compared with $NO_{3}^{-}$, which thereby alleviated Mn toxicity to rice. Acid soils are chemically dominated by a high $NH_{4}^{+}$/$NO_{3}^{-}$ ratio and an abundance of toxic Al and Mn (Zhao et al., 2014). In our previous studies, we found that $NH_{4}^{+}$ alleviated Al toxicity and reduced Al accumulation in rice and *Lespedeza bicolor* (Zhao et al., 2009; Chen et al., 2010; Wang et al., 2015a), while Al alleviated Mn toxicity and reduced Mn accumulation in rice (Wang et al., 2015b). In addition, Al-tolerant plants generally prefer $NH_{4}^{+}$ over $NO_{3}^{-}$ (Zhao et al., 2013; Zhao and Shen, 2018). The interactions of coexisting factors, such as $NH_{4}^{+}$, H^+^, and toxic Al and Mn, may thus facilitate the adaptation of plants to acid soils. This conclusion has important ecological implications for explaining the superior growth of plants in acid soils, where multiple stressful factors often coexist (Zhao et al., 2014).

### $NH_{4}^{+}$ Decreased Mn Accumulation by Reducing Mn Uptake Rather Than Mn Distribution in Leaves

The observed decrease in Mn accumulation in rice caused by low pH due to $NH_{4}^{+}$ uptake relative to $NO_{3}^{-}$ may result from the reduced ability of roots to take up Mn from nutrient solutions and/or the decreased ability of rice plants to translocate Mn from roots to leaves. In the present study, although low pH increased Mn distribution ratio in roots, neither low pH nor the form of N affected the distribution of Mn in different rice leaves. This observation suggests that the low pH-decreased Mn accumulation phenomenon in rice can be mainly attributed to a decrease in root Mn uptake ability rather than decreased Mn distribution in leaves. Mn toxicity to plants results mainly from high Mn accumulation in shoots, and roots are much less sensitive to Mn toxicity (Horiguchi, 1987). We propose that $NH_{4}^{+}$ decreases the amount of Mn taken up by rice roots because of the low pH, with less Mn consequently translocated to shoots, thereby alleviating Mn toxicity to rice.

### Cell Wall Properties Were Not Related With the $NH_{4}^{+}$-Reduced Mn Accumulation

The decreased Mn accumulation in rice roots caused by low pH may be attributed to low Mn adsorption in root cell walls and/or low Mn transport across the plasma membrane into root cells. In addition, one of the most important mechanisms of plant tolerance to metal toxicity is preventing the entry of metal ions into root cells. The cell wall usually plays an important role in plant defensive responses to metal stress (Krzesłowska, 2011). The negatively charged polymers of cell walls are responsible for interactions with exchangeable cations in the external medium (Richter and Dainty, 1990). The polysaccharides pectin and hemicellulose in the cell wall are major sites of cation accumulation because of their negative charges (Krzesłowska, 2011; Yang et al., 2011). In the present study, the Mn concentration in root cell walls was unaffected by either pH or the form of N when 5 mM Homo-PIPES pH buffer was added. Moreover, no difference was observed in the uronic acid contents of pectin and hemicellulose in cell walls extracted from rice roots between $NH_{4}^{+}$ and $NO_{3}^{-}$ in a non-pH-buffered solution (**Figure S1**). We found that Mn concentrations in root cell sap and xylem sap were lower at a pH of 3.5 than at pH 5.5, regardless of the N form, which suggests that low pH inhibited Mn transport across the plasma membrane into root cells. In our previous study, we demonstrated that altered cell wall properties, including pectin and hemicellulose contents, are responsible for $NH_{4}^{+}$-reduced Al accumulation in rice roots (Wang et al., 2015a). However, cell wall properties were not associated with the $NH_{4}^{+}$-reduced Mn accumulation in rice roots observed here. This decrease in Mn accumulation due to low pH may be attributed to the decrease in the amount of Mn entering cells.

### $NH_{4}^{+}$ Reduced Mn Uptake by Down-Regulating the Mn Transporter, OsNramp5

Two plasma membrane-localized Mn transporters, Nramp5 and MTP9, have been identified in rice roots (Ishimaru et al., 2012; Sasaki et al., 2012; Ueno et al., 2015). Nramp5 and MTP9 are located on the distal and proximal sides, respectively, of both the exodermis and endodermis of rice roots (Sasaki et al., 2012; Ueno et al., 2015). Nramp5 is correspondingly an influx Mn transporter and functions in transporting Mn from the soil solution to root exodermal and endodermal cells (Sasaki et al., 2012), while MTP9 is an efflux Mn transporter required for release of Mn from these cells towards the root stele (Ueno et al., 2015). Consistent with previous reports (Sasaki et al., 2012; Ueno et al., 2015), we found that the expression levels of both *Nramp5* and *MTP9* were not regulated by different Mn concentrations. The decreased pH due to $NH_{4}^{+}$ uptake, i.e., from pH 4.5 to 3.5, inhibited the expression of *Nramp5* but not *MTP9*, whereas the N form itself had less of an effect on the expression of either gene. Consequently, the decreased Mn accumulation in rice plants caused by low pH due to $NH_{4}^{+}$ uptake was associated with the down-regulation of expression of *Nramp5* rather than *MTP9*.

A previous study found that Si-decreased Mn uptake and toxicity result from the down-regulation of *OsNramp5* expression (Che et al., 2016). Similarly, Si reduces Cd accumulation and toxicity in rice by suppressing *OsNramp5* gene expression and protein levels (Shao et al., 2017), because *OsNramp5* can also transport Cd (Ishimaru et al., 2012; Sasaki et al., 2012). In the present study, the lower pH reduced Mn accumulation and *OsNramp5* expression level in rice, while Mn concentrations had no effect on the gene expression. This result suggests that the down-regulation of *OsNramp5* expression was caused by low pH itself rather than low pH-induced Mn changes. The exact mechanism underlying the down-regulation of expression of *OsNramp5* caused by low pH remains to be investigated in the future.

### The Up-Regulation of OsIRT1 May Contribute to Increased Zn Uptake at High pH

One previous report showed that excessive $NO_{3}^{-}$ supply up-regulated the expression of OsIRT1, which may be associated with increased medium pH by $NO_{3}^{-}$ uptake as nutrient solution was not pH-buffered (Yang et al., 2016). The present study showed that the expression level of OsIRT1 was also up-regulated at pH 5.5 relative to pH 3.5. This up-regulation may not contribute to the higher Mn uptake by rice at pH 5.5 than at pH 3.5, because previous research suggested that the role of OsIRT1 in Mn uptake by rice is negligible (Sasaki et al., 2012). Both OsNramp5 and OsIRT1 can transport Fe in rice (Bughio et al., 2002; Ishimaru et al., 2006; Ishimaru et al., 2012; Sasaki et al., 2012). Although high pH up-regulated the expression level of OsNramp5 and OsIRT1, high pH increased the Fe concentration of only roots but not shoots here. It is known that OsNramp5 and OsIRT1 are involved in the uptake of Fe^2+^ (Ishimaru et al., 2006; Ishimaru et al., 2012; Sasaki et al., 2012). Fe(III)-EDTA was supplied in this study, and this may explain why the up-regulation of OsNramp5 and OsIRT1 did not increase shoot Fe concentration. The increased Fe concentration of roots at high pH might be associated with other reasons such as immobilized Fe in roots. It is interesting to further investigate the effect of N forms and pH on the uptake of Fe^2+^ and Fe^3+^ by rice and its underlying molecular mechanism in the future. In addition, it was reported that over-expression of OsIRT1 increased Zn accumulation in rice (Lee and An, 2009). In agreement with the expression of OsIRT1, the concentration of Zn in shoots was increased by high pH in the present study. And this indicated that the up-regulation of OsIRT1 may contribute to increased Zn uptake at high pH. The inconsistency of OsIRT1 expression with Cu concentration of roots and shoots here suggested that OsIRT1 might not be involved in Cu transport.

## Conclusion

Our results indicate that $NH_{4}^{+}$ decreases Mn accumulation and toxicity in rice relative to $NO_{3}^{-}$, and this decrease is caused by a $NH_{4}^{+}$ -decreased pH but not by $NH_{4}^{+}$ itself. Low pH-decreased Mn accumulation in rice results from the decreased ability of roots to take up Mn because of the down-regulation of expression of the Mn influx transporter *OsNramp5*. In addition, high pH up-regulated the expression of *OsIRT1* and increased Zn uptake by rice.

## Data Availability Statement

All datasets [GENERATED/ANALYZED] for this study are included in the manuscript and the supplementary files.

## Author Contributions

AH and XZ conceived and designed the experiments. AH performed most of the experiments. MZ and LS conducted the collection and determination of root cell sap and xylem sap. AH, XZ, and RS analyzed data, prepared the figures, and wrote the manuscript. All authors contributed to manuscript revision, read and approved the submitted version.

## Funding

This work was supported financially by the National Natural Science Foundation of China (No. 31672229), the Key Research Program of the Chinese Academy of Sciences (KFZD-SW-112-01-09), the Research Startup Foundation of Nantong University (No. 03081206), and the Strategic Priority Research Program of the Chinese Academy of Sciences (No. XDB15030202).

## Conflict of Interest

The authors declare that the research was conducted in the absence of any commercial or financial relationships that could be construed as a potential conflict of interest.
