# Supplementary material for: Ammonium Alleviates Manganese Toxicity and Accumulation in Rice by Down-Regulating the Transporter Gene OsNramp5 Through Rhizosphere Acidification
Source: Front Plant Sci. 2019 Oct 3;10:1194. doi: 10.3389/fpls.2019.01194 (PMC6785973; doi:10.3389/fpls.2019.01194)
Supplement: Supplementary file 1 [file Presentation_1.pptx]

## Slide 1
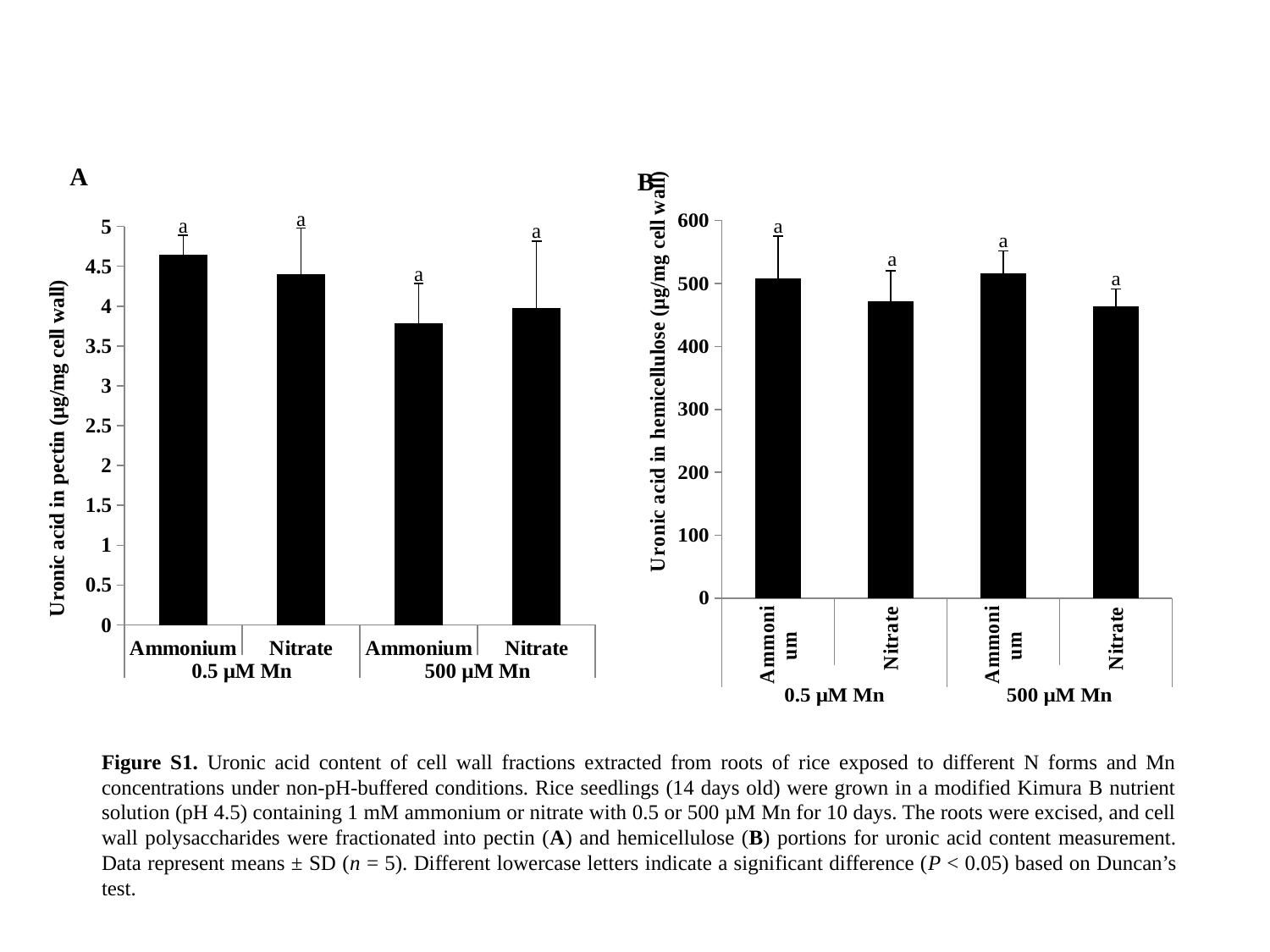

### Chart
| Category | 半纤维素 |
|---|---|
| Ammonium | 506.97237622820614 |
| Nitrate | 471.54403648160934 |
| Ammonium | 515.8884547763824 |
| Nitrate | 462.6803655225716 |A
B
### Chart
| Category | pectin |
|---|---|
| Ammonium | 4.64369555105447 |
| Nitrate | 4.394786336792226 |
| Ammonium | 3.781123077884929 |
| Nitrate | 3.9746157758376603 |Figure S1. Uronic acid content of cell wall fractions extracted from roots of rice exposed to different N forms and Mn concentrations under non-pH-buffered conditions. Rice seedlings (14 days old) were grown in a modified Kimura B nutrient solution (pH 4.5) containing 1 mM ammonium or nitrate with 0.5 or 500 µM Mn for 10 days. The roots were excised, and cell wall polysaccharides were fractionated into pectin (A) and hemicellulose (B) portions for uronic acid content measurement. Data represent means ± SD (n = 5). Different lowercase letters indicate a significant difference (P < 0.05) based on Duncan’s test.
